# Apocynin attenuates angiotensin II-induced vascular smooth muscle cells osteogenic switching via suppressing extracellular signal-regulated kinase 1/2

**DOI:** 10.18632/oncotarget.13193

**Published:** 2016-11-08

**Authors:** Weijing Feng, Kun Zhang, Yu Liu, Jie Chen, Qingqing Cai, Yinyin Zhang, Mongheng Wang, Jingfeng Wang, Hui Huang

**Affiliations:** ^1^ Guangdong Provincial Key Laboratory of Malignant Tumor Epigenetics and Gene Regulation, Department of Cardiology, Sun Yat-Sen Memorial Hospital, Sun Yat-Sen University, Guangzhou, China; ^2^ Laboratory of RNA and Major Diseases of Brain and Heart, Sun Yat-Sen Memorial Hospital, Sun Yat-Sen University, Guangzhou, China; ^3^ Department of Cardiology, the People's Hospital of Guangxi Zhuang Autonomous Region, Nanning, China; ^4^ Department of Radiation Oncology, Sun Yat-sen Memorial Hospital of Sun Yat-sen University, Guangzhou, China; ^5^ Department of Medical Oncology, Sun Yat-sen University Cancer Center, Sun Yat-sen University, Guangzhou, China; ^6^ Department of Physiology, Georgia Regents University, Augusta, GA, USA

**Keywords:** vascular smooth muscle cells, osteogenic switching, vascular calcification, angiotensin II, apocynin

## Abstract

Vascular calcification (VC) is a significant risk factor for cardiovascular morbidity and mortality. We recently reported that apocynin had benefits for preventing cardiovascular diseases. However, whether apocynin could attenuate VC is unknown. Here, we investigated the role of apocynin in VC and its underlying mechanisms. 163 participants with high or normal ankle–brachial index (ABI) were enrolled in this study for analyzing the demographic and biochemical data. In vitro, vascular smooth muscle cells (VSMCs) were exposed to calcification medium containing b-glycerophosphate and angiotensin II (Ang II) for 24 hours. The results showed that serum level of Ang II was significantly increased in patients with high ABI (P<0.05). In cultured VSMCs, Ang II significantly exacerbated osteogenic switching. The expression of osteogenic phenotype markers, including bone morphogenetic protein 2 (BMP2), runt-related transcription factor 2 (Runx2) and osteopontin (OPN), were significantly upregulated, whereas contractile markers expression, including alpha smooth muscle actin (a-SMA) and smooth muscle 22 alpha (SM22a) were simultaneously downregulated. However, these effects were greatly attenuated by apocynin. Apocynin enhanced expression of a-SMA by 5.3%, and reduced expression of BMP2, Runx2, OPN by 3.37%, 0.61% and 3.07%, respectively. Furthermore, extracellular signal-regulated kinase 1/2 (ERK1/2) phosphorylation was upregulated by Ang II, and this effect was also reversed by apocynin. Intriguingly, pretreatment with U0126, an inhibitor of ERK1/2, had similar effects with apocynin. Apocynin may act as a novel molecular candidate to protect against VSMCs osteogenic switching through suppressing ERK1/2 pathway.

## INTRODUCTION

Vascular calcification (VC) has been demonstrated to be a common vasculopathy of atherosclerosis, chronic kidney disease, hypertension and diabetes [1–3]. It is directly associated with a high cardiovascular morbidity and mortality [4]. It has been shown that the prevalence of VC is estimated to be 40% to 99% in patients with chronic kidney disease, leading to a heavy burden on public health [5, 6]. Although a great effort has been made on studying VC, currently there still lacks of effective therapies available to treat or prevent the development of VC. Thus, to explore the mechanisms and look for new therapeutic strategies to prevent VC remains urgent.

The mechanisms contributing to VC are complicated [7, 8]. Vascular smooth muscle cells (VSMCs), the predominant cell type of arterial wall, are essential to maintain structural and functional integrity of vessels [9]. It has been considered that VSMCs switching from a contractile to an osteogenic phenotype plays an essential role in the process of VC [10–12]. Moreover, a series of factors, such as angiotensin II (Ang II), play important role in the development and progression of VC [13]. Recently, Zhang et al. reported an interesting finding that Ang II promoted phenotypic switching of VSMCs [14]. Nevertheless, it remains lack of effective therapies to improve ANG II-induced phenotypic switching.

Apocynin is an important bioactive substance of cardiovascular system that has been found to be involved in anti-inflammation, anti-hypertension, preventing vascular injury, etc [15–18]. Our recent study also found that apocynin plays an important role in attenuating cardiac injury and cardiac remodeling [19, 20]. However, whether it has an effect on regulating Ang II-induced VSMCs phenotypic switching and what is the cellular and molecular mechanism are still unknown. Extracellular signal-regulated kinase 1/2 (ERK1/2) pathway is responsible for conveying information about the extracellular environment to the cell nucleus and is known to positively regulate VC [21, 22]. But whether the ERK1/2 signaling is involved in the regulation of Ang II-induced VSMCs phenotypic switching has not yet been completely clarified. Thus, in the present study, we hypothesized that apocynin might improve Ang II-induced VC via attenuating VSMCs switching from a contractile to an osteogenic phenotype and this effect may be involved in ERK1/2 activation.

## RESULTS

### Comparison of demographic and biochemical data between participants with high and normal ABI

A total of 163 participants without potential infectious or inflammatory diseases, immunologic diseases, chronic kidney disease, hyperparathyroidism and carcinoma were enrolled in this study. According to the values of ABI, we grouped the participants into high ABI group (ABI≥1.3) and normal ABI group (0.9<ABI<1.3). The demographic and biochemical data of two groups are shown in Table 1. As shown in the table, patients with high ABI showed a significant increase in the serum level of Ang II compared with normal ABI group (52.77±11.71 ng/L vs 34.42±7.27 ng/L, P < 0.05, Table 1). Additionally, no statistically significant differences were observed in age, gender, Cr, UA, FPG, ALP, phosphate, calcium and serum lipid levels (P > 0.05, Table 1).

**Table 1:** Comparison of demographic and biochemical data between participants with high and normal ABI

	High ABI group	Normal ABI group	P value
Number (n)	64	99	−
Age (year)	57±11	56±10	0.394
Male gender (%)	59.9	60.9	0.874
ALP (U/L)	66(55-80)	64(54-73)	0.419
Ca (mmol/L)	2.26±0.38	2.29±0.15	0.109
P (mmol/L)	1.12±0.20	1.11±0.18	0.329
TC (mmol/L)	5.01±1.26	5.16±1.08	0.223
LDL-C (mmol/L)	3.06±0.90	3.12±0.90	0.133
HDL-C(mmol/L)	1.30±0.32	1.33±0.38	0.323
TG (mmol/L)	1.55(0.93-2.36)	1.54(0.99-2.13)	0.761
Apo A (g/L)	1.13(1.03-1.30)	1.18(1.06-1.37)	0.093
Apo B (g/L)	0.79±0.23	0.82±0.18	0.051
Cr (μmol/L)	96(86-110)	100(90-112)	0.270
UA (μmol/L)	398.73±104.12	397.04±104.12	0.903
FPG (mmol/L)	5.38(4.63-5.70)	5.24(4.60-5.80)	0.834
Ang II (ng/L)	52.77±11.71	34.42±7.27	0.001*

ABI, ankle–brachial index; ALP, alkaline phosphatase; Ca, calcium; P, phosphate; TC, total cholesterol; LDL-C, low-density lipoprotein cholesterol; HDL-C, high-density lipoprotein cholesterol; TG, triglycerides; Apo, apolipoprotein; FPG, fasting plasma glucose; Cr, creatinine; UA, uric acid; Ang II, angiotensin II; ABI, ankle-brachial index. Values are mean ± SD or median (interquartile range).

* P<0.05 vs. normal ABI group.

### The serum level of Ang II was positively associated with high ABI and VC

**Figure 1:**
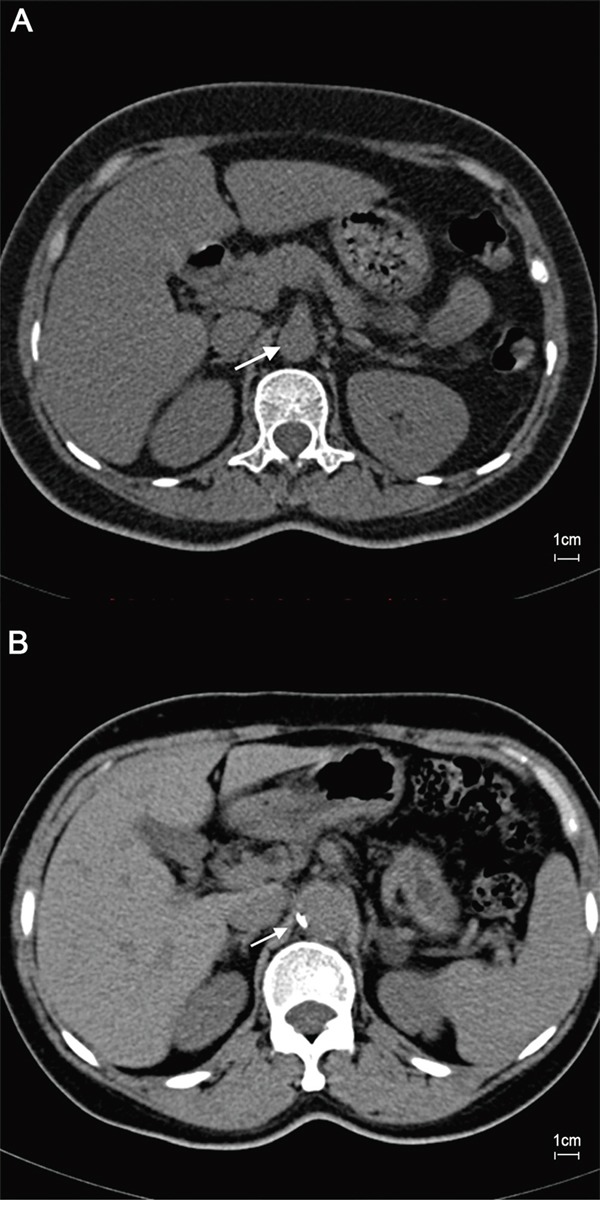
Axial cross-sections were obtained by computed tomography in patients with normal and high ABI The image in a 47-year-old patient with normal ABI showed no obvious calcification (arrow) in abdominal aorta **A.** However, abdominal aorta calcification was evident (arrow) in a 46-year-old patient with high ABI **B.** Scale bar=1cm.

VC is characterized by high ABI [23]. In agreement with previous studies, we also found that VC was significantly more pronounced in patient with high ABI compared with normal one (Figure 1). To further evaluate the association between Ang II and VC in participants, multivariate regression analysis was used to explore the correlations between high ABI and various risk factors. We found that Ang II was proved to be an independent risk factor of VC in patients with high ABI (Ang II, β=0.53, P=0.003) (Table 2).

**Table 2:** The multivariate regression analysis of the independent risk factors for high ABI

	β	P
Age	0.012	0.512
ALP	0.004	0.429
Ca	−1.528	0.219
P	0.624	0.411
TC	−0.051	0.619
TG	0.045	0.318
LDL-C	−0.127	0.371
HDL-C	−0.262	0.335
Apo A	−0.654	0.302
Apo B	−1.018	0.294
Cr	−0.023	0.081
UA	0.106	0.743
FPG	0.084	0.467
Ang II	0.531	0.003*

The multivariate regression analysis was used to assess the independent risk factors for high ABI patients in unadjusted model (*P < 0.05).

### Apocynin ameliorated VSMCs calcification induced by Ang II

**Figure 2:**
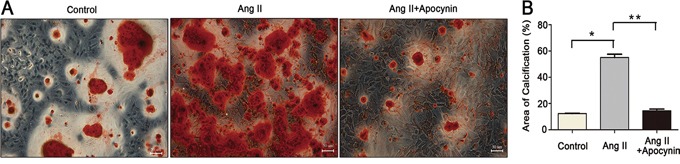
Apocynin attenuated Ang II-induced VSMCs calcification **A.** Representative Alizarin Red staining micrographs showed that the increased VSMCs calcification in Ang II group was attenuated by apocynin (scale bar=50μm). **B.** Bar graph showed the area of calcification. Data are expressed as mean±SD. n=4, *P<0.05 vs Control group; **P<0.05, ***P<0.05 vs Ang II group.

To identify the role of apocynin in VSMCs calcification, we performed the Alizarin Red staining in VSMCs. Calcification was significantly increased by the treatment with Ang II. However, this effect was greatly suppressed by the co-treatment with apocynin (Figure 2). These results indicated that apocynin may be a promising therapeutic agent for VSMCs calcification.

### Apocynin attenuated Ang II-induced VSMCs switching from a contractile to an osteogenic phenotype

**Figure 3:**
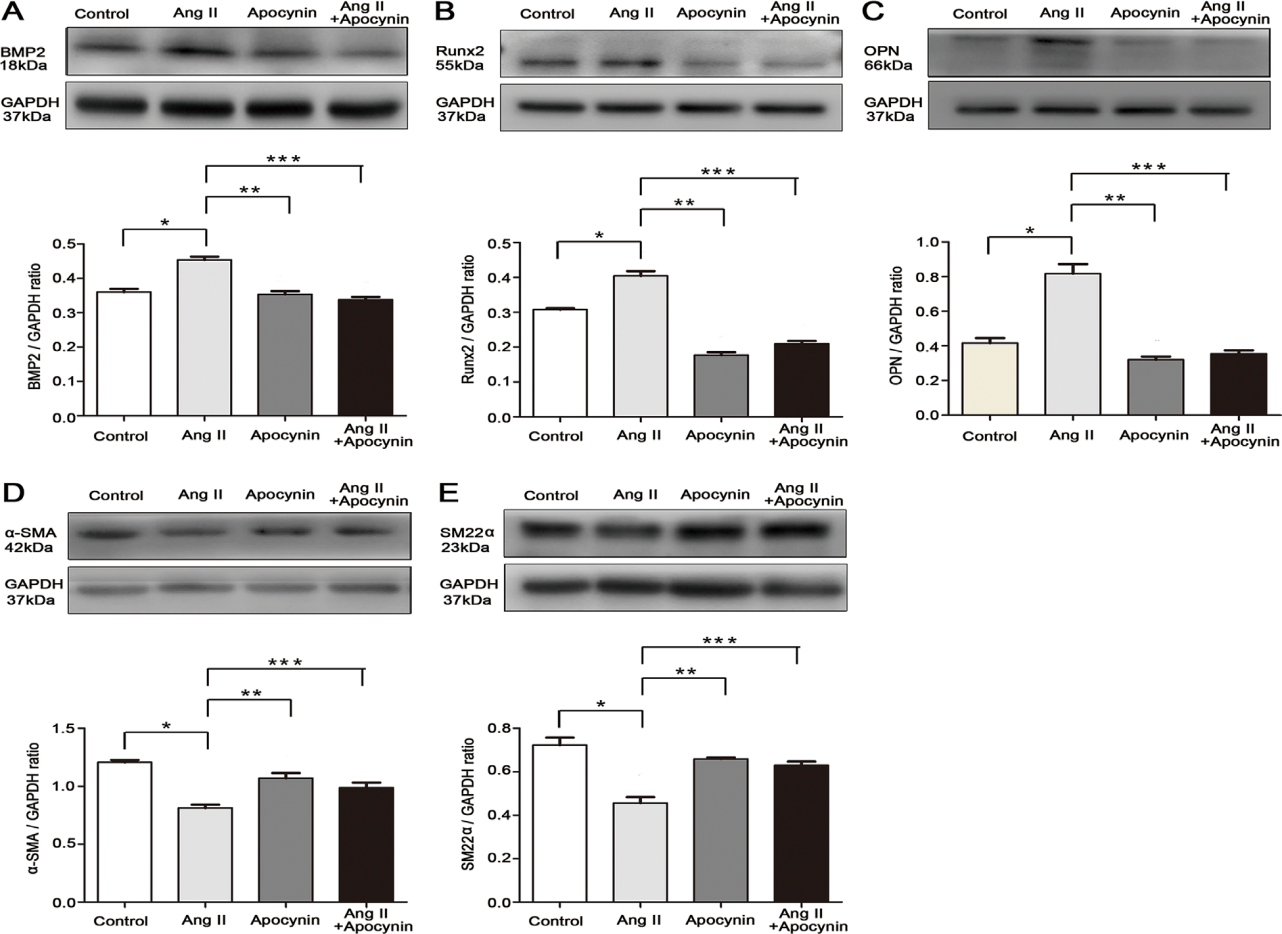
Apocynin inhibited VSMCs switching from contractile to osteogenic phenotype **A, B** and **C.** Western blot results showed that Ang II–induced upregulations of osteogenic phenotype markers, including BMP2, Runx2 and OPN, were reduced by apocynin (n=4). **D** and **E.** Apocynin ameliorated Ang II-induced downregulations of contractile phenotype markers, including α-SMA and SM22α. Data are expressed as mean±SD. n=4, *P<0.05 vs Control group; **P<0.05, ***P<0.05 vs Ang II group.

**Figure 4:**
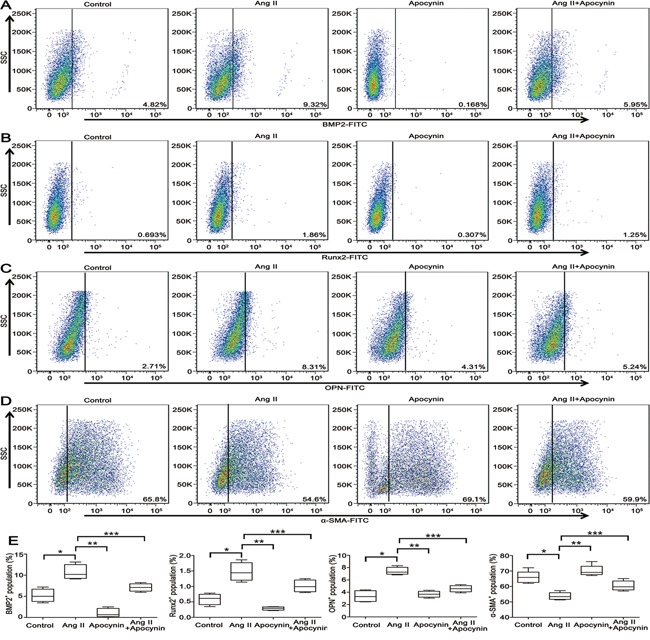
VSMCs switching from contractile to osteogenic phenotype was suppressed by apocynin **A, B** and **C.** Flow cytometry results confirmed that Ang II-induced the expression of osteogenic phenotypes, including BMP2, Runx2 and OPN, were reduced by apocynin. **D.** Apocynin increased the expression of contractile phenotype (α-SMA) as determined by western blot. **E.** The positive percentage of BMP2^+^, Runx2^+^, OPN^+^ and α-SMA^+^ VSMCs in each group was shown by box blots illustrating the median and inter-quartile range of positive VSMCs. n=5, *P<0.05 vs Control group; **P<0.05, ***P<0.05 vs Ang II group.

VSMCs switch into osteo-/chondrocytic-like cells and express osteogenic phenotypic protein during VC that has drawn more attention in recent years [24]. We attempted to investigate whether apocynin has the potential effect on preventing Ang II-induced VSMCs phenotype transdifferentiation. Stimulation of VSMCs with Ang II, the osteogenic switching markers, including bone morphogenetic protein 2 (BMP2), runt-related transcription factor 2 (Runx2) and osteopontin (OPN), were significantly increased compared with control (Figure 3A-3C). Simultaneously, the expression of contractile markers, alpha smooth muscle actin (α-SMA) and smooth muscle 22 alpha (SM22α), were markedly reduced by Ang II (Figure 3D-3E). Furthermore, flow cytometry analysis also showed that treatment with Ang II decreased α-SMA^+^ VSMCs by 11.2% at 24 hours, but increased the positive percentage of BMP2^+^, Runx2^+^ and OPN^+^ VSMCs by 4.5%, 1.167% and 5.6%, respectively (P<0.05; Figure 4). These results indicated that Ang II induced VSMCs switching from a contractile to an osteogenic phenotype in the process of VSMCs calcification. However, all the effects were markedly attenuated by the pretreatment with apocynin (Figure 3 and 4).

### ERK1/2 activation was inhibited by apocynin in VSMCs phenotypic switching

**Figure 5:**
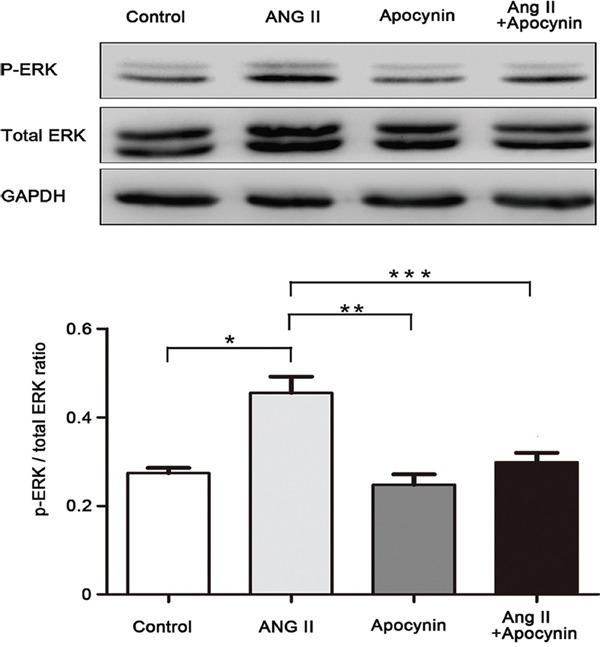
Apocynin inhibited Ang II-induced phosphorylation of ERK1/2 in VSMC Data are expressed as mean±SD. n=4, *P<0.05 vs Control group; **P<0.05, ***P<0.05 vs Ang II group.

**Figure 6:**
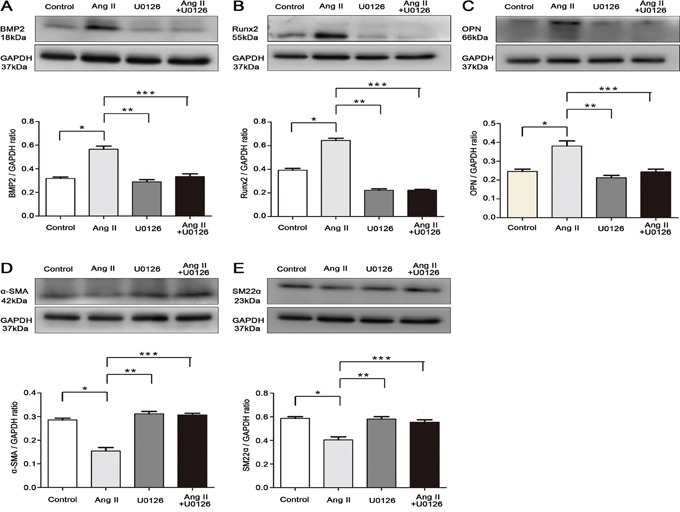
Apocynin attenuated VSMCs calcification via inhibiting ERK1/2 activation and osteogenic phenotype switching **A, B** and **C.** Ang II–induced expression of osteogenic phenotype markers, including BMP2, Runx2 and OPN, were reduced by U0126 (ERK1/2 inhibitor) in VSMCs. **D** and **E.** U0126 involved in inhibiting Ang II-induced downregulations of α-SMA and SM22α. Data are expressed as mean±SD; n=3 for each group. *P<0.05 vs Control group; **P<0.05, ***P<0.05 vs Ang II group.

**Figure 7:**
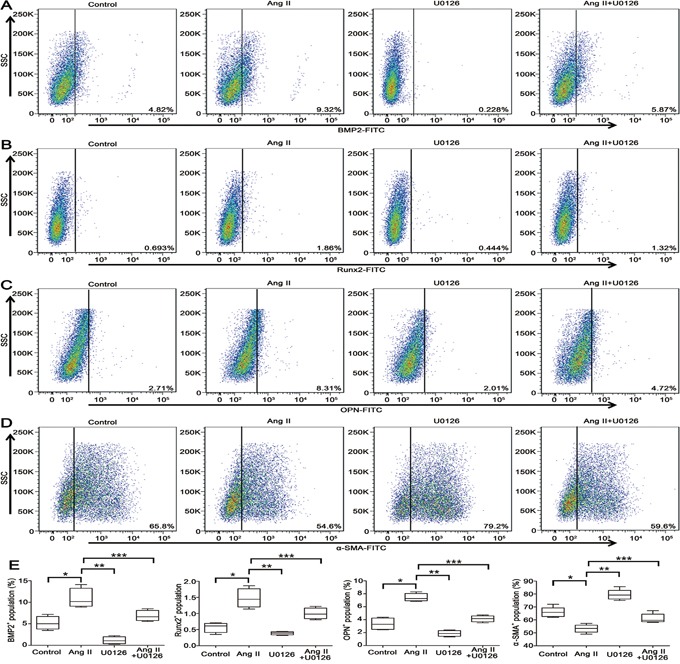
VSMCs switching to osteogenic phenotype involved in ERK1/2 inhibition by U0126 **A, B** and **C.** Flow cytometry indicated that U0126 suppressed the expression of osteogenic phenotypes, including BMP2, Runx2 and OPN. **D.** Ang II-induced downregulations of α-SMA were inhibited by U0126. **E.** The percentage of BMP2^+^, Runx2^+^, OPN^+^ and α-SMA^+^ VSMCs in each group is shown by box blots illustrating the median and inter-quartile range of positive VSMCs. n=5, *P<0.05 vs Control group; **P<0.05, ***P<0.05 vs Ang II group.

We further explored whether ERK1/2 activation was involved in the beneficial effects of apocynin during the process of VSMCs phenotypic switching. We found that Ang II significantly induced phosphorylation of ERK1/2 in the process of VSMCs osteogenic switching. However, this effect was significantly inhibited by apocynin (Figure 5). Furthermore, pretreatment with ERK1/2 inhibitor U0126 prevented Ang II-induced upregulation of BMP2, Runx2 and OPN, and increased the expression of α-SMA and SM22α (Figure 6). Additionally, flow cytometry results also showed that U0126 treatment improved Ang II induced VSMCs osteogenic switching. It was found that the expression of contractile marker (α-SMA) was significantly increased by 5%, whereas the osteogenic markers (BMP2, Runx2, OPN) were greatly decreased by 3.45%, 0.54% and 3.59% at 24 hours, respectively, compared with Ang II group (P<0.05; Figure 7). These results indicated that apocynin played a protective effect against VSMCs calcification and phenotypic switching might via inhibiting ERK1/2 activation.

## DISCUSSION

The present study explored the role of apocynin during the process of Ang II-induced VSMCs osteogenic switching and calcification. We found that the serum level of Ang II was remarkably elevated in patients with high ABI whom often accompanied with VC. In addition, we showed that Ang II exacerbated VSMCs osteogenic phenotypic switching in vitro. Furthermore, our findings demonstrated that apocynin attenuated Ang II-induced VSMCs switching from a contractile to an osteogenic phenotype possibly through inhibition of ERK1/2 activation.

According to the clinical data, we found that Ang II was significantly increased in patients with high ABI. These evidences indicated that elevated Ang II has a close correlation with increased risk of VC. To gain further insight into the mechanisms how Ang II promoted VC, we explored the phenotypic switching in VSMCs. In accordance with previous studies [13], we found that Ang II could induce VSMCs to undergo phenotypic switching characterized by reducing expression of contractile markers (α-SMA, SM22α) and upregulated osteogenic markers (BMP2, Runx2, OPN). Additionally, we also noticed that, Ang II markedly accelerated the progression of VSMCs calcification compared with the treatment with β-glycerophosphate alone for 24 hours. These findings importantly implied that Ang II was an important risk fact for VC.

Apocynin had many beneficial effects on cardiovascular disease, including anti-inflammation, reducing endothelial activation, protecting vascular injury, etc [25, 26]. In the present study, we found that apocynin played a protective role in VSMCs osteogenic switching. Since inflammation and oxidative stress have been implicated in the development of VC, the protective action of apocynin in VSMCs osteogenic switching may be associated with its biological properties. However, the potential mechanisms are not clear. Although Brodeur et al found that downregulating the osteogenic mRNA expression of cbfa1, alkaline phosphatase and osteocalcin was associated with the beneficial effects of apocynin on VC [27], the mechanisms between apocynin and VSMCs phenotypic switching have not been further explored. In this work, we paid more attention to how apocynin affected VSMCs switching from a contractile to osteogenic phenotype. We noted that apocynin could improve the development of VSMCs calcification, and this effect was characterized by upregulation the protein level of contractile phenotype markers (α-SMA, SM22α) as well as downregulation of osteogenic phenotype markers (BMP2, Runx2, OPN). Such observations have demonstrated that apocynin could improve VC via inhibiting VSMCs osteogenic phenotypic switching, and this effect may be a novel target for apocynin ameliorating progression of calcification.

**Figure 8:**
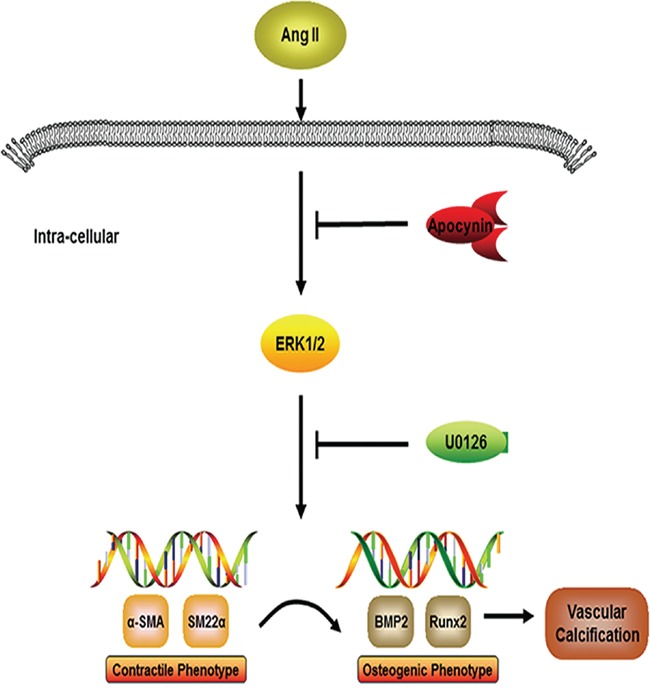
The potential mechanisms for apocynin to attenuate vascular calcification induced by ANG II Apocynin improves vascular calcification induced by Ang II. VSMCs switching from a contractile to an osteogenic phenotype, a crucial process of vascular calcification, are suppressed by apocynin through inhibiting ERK1/2 signaling pathway.

To explore the cellular and molecular mechanisms underlying VSMCs osteogenic switching improved by apocynin, we explore the ERK1/2 signaling. We focused on this pathway for three main reasons: (1) Ang II significantly enhanced the ERK phosphorylation levels [28, 29]; (2) Our and previous studies demonstrated that apocynin could interfere with the phosphorylation of ERK1/2; [20] and (3) ERK1/2 activation has been implicated to positively regulate VC [30]. In the present study, we demonstrated for the first time that apocynin significantly suppressed the phosphorylation of ERK1/2 in rat VSMCs calcification induced by Ang II. In order to examine our hypothesis, we further found that inhibition of ERK1/2 with U0126 decreased expression of osteogenic markers and increased VSMCs contractile markers. These findings indicated that ERK1/2 pathway might act as a bridge between contractile and osteogenic phenotype switching by which apocynin attenuated VSMCs calcification induced by Ang II (Figure 8). Whether other mechanisms are involved in apocynin ameliorating VSMCs calcification and phenotypic switching needs further study.

There are several limitations in this work. First, in this study, Ang II receptor blockers (ARBs) were not used as positive control treatment. Armstrong ZB et al have demonstrated that ARBs might improve VC [31]. However, this model induced by atherogenic diet was different from VSMCs calcification or media calcification. We will compare the different and synergistic effects of apocynin and ARBs on VC in further studies. Also, in vitro, the Ang II induced VSMCs calcification model cannot completely mimic the condition in VC, since a series of mechanisms are disorder in VC. Besides, the action of apocynin in vivo has not been further explored.

In conclusion, the present study demonstrated that Ang II exacerbated VSMCs calcification and switching from a contractile to an osteogenic phenotype. These processes were attenuated by apocynin treatment. Blocking phosphorylation of ERK1/2 with U0126 improved the osteogenic phenotypic switching in VSMCs, which suggested that ERK1/2 inhibition was involved in the beneficial effects of apocynin. These findings provide new insights into the important roles of apocynin in cardiovascular system, and it suggests that apocynin may use as a promising therapeutic target for vascular calcification.

## MATERIALS AND METHODS

### Study population and data collection

Between September 2012 and November 2014, all 163 participants who came to the Sun Yat-sen Memorial Hospital for either routine physical examinations or hospitalizations were enrolled in this study. All these participants underwent ankle–brachial index (ABI) test and were studied by an anonymous way. The patients were excluded as described previously [32]. Among these 163 participants, 64 participants had ABI≥1.3, and were defined as the high ABI group. And the other 99 participants with normal ABI (0.9<ABI<1.3) were defined as the normal ABI group [23]. The study protocol conformed to the ethical guidelines of the 1975 Declaration of Helsinki. For the use of these clinical materials for the research, prior patient's consent and approval from the Institute Research Ethics Committee of Sun Yat-sen Memorial Hospital of Sun Yat-sen University were obtained.

Blood samples were obtained by venipuncture after ≥10 hours of overnight fasting. Serum levels of uric acid (UA), alkaline phosphatase (ALP), creatinine (Cr), calcium (Ca), phosphate (P), total cholesterol (TC), triglycerides (TG), high-density lipoprotein cholesterol (HDL-C), low-density lipoprotein cholesterol (LDL-C), apolipoprotein A (apoA), apolipoprotein B (apoB) and fasting plasma glucose (FPG) were measured by a standardized and certified program with an automatic biochemical analyzer (7170A, HITACHI, Japan). Serum Ang II level was tested with radioimmunoassay kits (Beijing North Institute of Biological Technology, Beijing, China).

### Measurement of ABI

Measurement of ABI was performed with a non-invasive vascular screening device (VP-1000, OMRON, Japan) as described in our previous study [33]. The systolic blood pressure (SBP) measurements in the posterior tibial and dorsal pedal arteries of both legs and brachial artery of both arms were obtained. The ABI for each leg was calculated by dividing the highest posterior tibial or dorsal pedal SBP by the highest arm SBP. ABI was a maneuverable, noninvasive and reliable method for indicating suspicious VC [34].

### Computed tomography

Measurement of abdominal aorta calcification (AAC) was performed on a 64-row computed tomography scanner (Sensation 64, Siemens Medical Solutions, Erlangen, Germany) as previously described [35]. The consecutive axial cross-sections of abdominal aorta were acquired with a slice thickness of 3 mm. To assess of the amount and quantity of AAC, the images were transferred to a workstation VITREA2 (Vital Images, Inc.) equipped with software to calculate Agatston score. The regions of interest around identified calcification were evaluated by an experienced radiologist in a blinded fashion. Then, the total number of pixels within the regions of interest was calculated for Agatston score of AAC with semiautomatic software.

### Cell cultures and treatment

The animal care and experimental protocols were approved by the Committee on Ethics of Animal Experiments and conducted in accordance with the Guidelines for Animal Experiments, Sun Yat-sen University and the Guide for the Care and Use of Laboratory Animals published by the US National Institutes of Health (NIH Publication No. 85-23, revised 1996). Primary aortic VSMCs of 2-month-old male Sprague–Dawley rats were obtained as described previously [36] and maintained in the high glucose (4.5g/L) Dulbecco's modified Eagle's medium (DMEM) containing 10% fetal bovine serum (FBS), 100 U/ml penicillin and 100 mg/ml streptomycin (Invitrogen Life Technologies) at 37°C in a humidified atmosphere containing 5% CO_2_. VSMCs at passages 4 to 8 were used for experiment. VSMCs at 80% confluence were shifted to the calcification media containing 10% FBS, 10 mM sodium pyruvate, 100 U/ml penicillin, 100 mg/ml streptomycin and 10 mM β-glycerophosphate (Sigma) for 7 days with media changes every 2 days.

The cells were cultured in a serum-free maintenance DMEM medium for 24 hours before use. Then, the cells were shifted to the DMEM medium with or without β-glycerophosphate and treated with (a) dimethyl sulfoxide (DMSO, 1ul, Sigma), (b) Ang II (100nM; Sigma), (c) apocynin (100μM; Sigma), (d) Ang II (100nM) plus apocynin (100μM), (e) ERK1/2 inhibitor U0126 (10μM; Cell signaling technology) and (f) Ang II (100nM) plus U0126 (10μM). Ang II was dissolved with phosphate buffered solution (PBS). Both apocynin and U0126 were dissolved in DMSO and added 1 hour before the stimulation of Ang II. The protein expression of bone morphogenetic protein 2 (BMP2), runt-related transcription factor 2 (Runx2), osteopontin (OPN), alpha smooth muscle actin (α-SMA) and smooth muscle 22 alpha (SM22α) were detected after the treatment with Ang II for 24 hours. The phosphorylation of ERK1/2 was detected after the incubation with Ang II for 15 minutes. All of the experiments were performed in triplicate.

### Alizarin red staining

After treatment, cells were washed 3 times with PBS and then fixed with 4% paraformaldehyde for 45 minutes. Then, the cells were washed 3 times with PBS and exposed with 2% Alizarin Red solution (Sigma) for 10 minutes. After washing with PBS 3 times, cells were photographed with microscope. Positively stained cells display a red-orange color.

### Western blot analysis

The protein samples were extracted from VSMCs with RIPA lysis buffer (Beyotime, Haimen, China) on ice for 30 min. The process of western blot was used as previously described [37]. The following primary antibodies were used: anti-total ERK1/2 antibody (#9926, Cell signaling technology), anti-phospho-ERK1/2 antibody (#9910, Cell signaling technology), anti-GAPDH antibody (#2118, Cell signaling technology), anti-BMP2 antibody (sc-6895, Santa Cruz), anti-Runx2 antibody (sc-10758, Santa Cruz), anti-osteopontin antibody (ab8448, Abcam), anti-α-SMA antibody (ab8211, Abcam), anti-SM22α antibody (ab10135, Abcam). The bands were analyzed semi-quantitatively.

### Flow cytometry

VSMCs were harvested by centrifugation, washed, and resuspended in PBS. Cells were then fixed with fixation medium (GAS003, Invitrogen Life Technologies) for 15 min at room temperature before adding primary antibody. After washing with PBS, cells were incubated with permeabilization medium (GAS003, Invitrogen Life Technologies) and primary antibody or the corresponding isotype-matched IgG control (BD Biosciences) for 30 min in the dark at room temperature. Cells stained for BMP2, Runx2 or osteopontin were washed twice and incubated with FITC-conjugated anti-mouse secondary antibody (554001, BD Biosciences) or FITC-conjugated anti-rabbit secondary antibody (sc-2090, Santa Cruz). The cells were subjected to flow cytometric analysis using FACSVerse (BD Biosciences), and data were analyzed with FlowJo software.

### Statistical analysis

Normal distribution data are expressed as mean ± standard error (SE), and non-normal distribution data are presented as median with interquartile range. Comparisons between two groups were analyzed by t-test or Mann-Whitney U test. Differences of data among more than three groups were determined by One-way ANOVA followed by a Bonferroni comparison test. Multivariate regression analysis was used to assess the independent risk factors for high ABI. Statistical analysis was performed with SPSS version 17.0 (SPSS Inc.). Values of P<0.05 were considered statistically significant.
